# Evaluation of hackberry (*Celtis* *australis* L.) fruits as sources of bioactive compounds

**DOI:** 10.1038/s41598-023-39421-x

**Published:** 2023-07-28

**Authors:** Farkhondeh Safari, Hamid Hassanpour, Ahmad Alijanpour

**Affiliations:** 1 Department of Horticultural Sciences, Faculty of Agriculture, Urmia University, Urmia, Iran; 2 Department of Forestry, Faculty of Natural Resources, Urmia University, Urmia, Iran

**Keywords:** Biochemistry, Physiology, Plant sciences

## Abstract

Hackberry (*Celtis* *australis* L.) is native to the Mediterranean region and is distributed in Europe, Turkey, North Africa, and Iran. To the best of our knowledge, no study has been conducted on *C.* *australis* L. in the Arasbaran region, Iran. In the present study, total phenol (TP), flavonoid (TF), antioxidant capacity based on DPPH and FRAP assays and phenolic compounds and sugars profiles were investigated. According to the results, the range of antioxidant capacity based on DPPH and FRAP assays was 14.12–88.24% and 44.35–117.87 mg Fe^2+^/100 g, respectively. Also, the range of gallic acid, caffeic acid, chlorogenic acid, rutin, *p*-coumaric acid, rosmaric acid, cinnamic acid, and apigenin content was 2.59–26.32, 2.03–9.32, 0.94–11.35, 1.80–4.857, 2.32–9.52, 4.74–51.38, 0.18–2.10 and 0.27–1.37 mg/g, respectively. The results of factor analysis showed that the C12, C14, C15, C20, C8, C16, C3, and C20 genotypes are positively characterized by the first principal component (PCA1) that have a higher caffeic acid, chlorogenic acid, rutin, *p*-coumaric acid, rosmaric acid, quercetin, cinnamic acid, and apigenin phenolic compounds. Based on cluster analysis, the twenty genotypes were located in 2 main clusters. In general, the obtained results can be useful for breeding programs and the introduction of cultivars in *Celtis* *australis* L.

## Introduction

Hackberry (*Celtis* *australis* L.) belongs to the Cannabaceae family, which is native to the Mediterranean region and is distributed in Europe, Turkey, North Africa, and Iran^1^. In Iran, it is distributed in the Alborz and Zagros mountains and mixed with the oak communities^2^. Hackberry is a deciduous tree with a height of 15–20 m^3^. Hackberry fruit is a drupe and ovoid and ripens in autumn and the colour of ripe fruit is yellow, brown, and black^4^. *Celtis* species are known as medicinal plants due to the presence of secondary metabolites such as flavonoids, steroids, terpenoids, and tannins. Previous studies in *Celtis* resulted in the isolation of phenolic glycosides^5^, steroids^6^, terpenoids^7^, tannins, saponins, and alkaloids^8^.

The fruit and leaf of hackberry trees contain phenolic compounds, flavonoids, tannins, antimicrobial, antifungal compounds, vitamins, tocopherol, carotenoids, minerals, fiber, protein, and fatty acids^4^. Previous studies have shown that hackberry fruit is a rich source of phytochemicals such as phenolic compounds, flavonoids, and minerals. Currently, the interest in hackberry fruit as one of the main sources of antioxidants has increased^4^.

In a previous study, it was found that the total phenol content in ripe fruit was 0.27 g gallic acid equivalent (GAE)/100 g and its main phenolic compound was Cyaniding-3,5-di-o-glucoside^4^. Filali**-**Ansari et al.^9^ reported that flavonoids are an important group in *C.* *australis* and its level in fruits was 34.14 mg catechin (CAT)/g DW and fruit tannin content was 5.69 mg CAT/g DW. The presence of these compounds in the *C.* *australis* fruits resulted in biological activities such as anti-inflammatory, antimicrobial, and antioxidant properties. Also, Dior Fall et al.^10^ found that coumarins, flavonoids, mucilages, terpenoids, and steroids were the major phytochemical compounds in *C.* *australis*.

Furthermore, Filali**-**Ansari et al*.*^9^ examined the antioxidant activity of *C.* *australis* fruit and reported that inhibition of the free radical scavenging activity of DPPH, thiobarbituric acid inhibitors and inhibition of malondialdehyde during linoleic acid peroxidation at concentrations of 2, 10, and 20 mg/ml were 64, 85 and 92%, respectively. Moreover, under the same conditions, the inhibition of TBARS activity in fruit was 55, 78, and 81%, respectively. Besides that, in this study, the total phenol content of *C.* *australis* L. fruits was reported 286.276 mg GAE/g FW.

Hackberry fruit is a very rich source of phytochemicals such as phenolic compounds, flavonoids, organic acids and minerals. Therefore, considering that hackberry fruit has food and medicinal uses in most countries such as Iran, Turkey, etc., as a result, its production can be effective in human health. Hence, since no study has been conducted on the phytochemical properties, antioxidant capacity, and phenolic compounds and sugar profiles of wild hackberry genotypes in the Arasbaran region of East Azerbaijan province, Iran, it seems necessary to conduct such basic studies on wild hackberry genotypes. Therefore, this study aimed to investigate and compare the antioxidant properties, and phenolic compounds and sugars profiles of twenty wild *C.* *australis* L. genotypes grown in Iran. The obtained results could be used for cultivation or breeding programs of *C.* *australis* L. and in food industries.

## Materials and methods

In this study, twenty *C.* *australis* L. genotypes were selected from the Arasbaran region of East Azerbaijan province, Iran according to free of pest and disease characteristics and labeled (Table 1). The genotypes were identified and authenticated by Department of Forestry in Urmia University and voucher specimens (NO. 1504) were deposited at the herbarium of the horticultural department, Urmia University, Urmia, Iran. The fruits were harvested from different directions of the tree at full maturity and then transferred to the lab for measuring different parameters. All used chemicals were analytical degree (Sigma-Aldrich Company, St. Louis, MO, USA).

**Table 1:** The altitude, latitude, longitude and climatic conditions where the genotypes were collected.

G	Collected site	Altitude (m)	Latitude	Longitude	Mean annual	Mean annual	Average of relative humidity (%)
					Temperature (°C)	Precipitation (mm)	
C1	Khodaafarin	1460	39° 2ʹ 35.1″ N	47° 57ʹ 19.3″ E	14.1	395.3	64
C2	Khodaafarin	1428	39° 2ʹ 31.64″ N	47° 57ʹ 14.7″ E	14.1	395.3	64
C3	Khodaafarin	1440	39° 2ʹ 31.56″ N	47° 57ʹ 13.5″ E	14.1	395.3	64
C4	Khodaafarin	1461	39° 2ʹ 30.49″ N	47° 57ʹ 9.63″ E	14.1	395.3	64
C5	Khodaafarin	1454	39° 2ʹ 27.34″ N	47° 57ʹ 6.34″ E	14.1	395.3	64
C6	Khodaafarin	1359	39° 2ʹ 27.92″ N	47° 57ʹ 4.32″ E	14.1	395.3	64
C7	Khodaafarin	1420	39° 2ʹ 27.31″ N	47° 57ʹ 4.01″ E	14.1	395.3	64
C8	Khodaafarin	1465	39° 2ʹ 27.02″ N	47° 57ʹ 4.09″ E	14.1	395.3	64
C9	Khodaafarin	1423	39° 2ʹ 25. 16″ N	47° 57ʹ 1.12″ E	14.1	395.3	64
C10	Khodaafarin	1482	39° 2ʹ 26.26″ N	47° 57ʹ 1.11″ E	14.1	395.3	64
C11	Kalibar	1487	38° 2ʹ 31.05″ N	46° 57ʹ 11.02″ E	12.7	401.9	62
C12	Kalibar	1429	38° 2ʹ 31.33 ″ N	46° 57ʹ 11.56″ E	12.7	401.9	62
C13	Kalibar	1410	38° 2ʹ 42.03″ N	46° 57ʹ 12.24″ E	12.7	401.9	62
C14	Kalibar	1412	38° 3ʹ 50.08″ N	46° 57ʹ 7.80″ E	12.7	401.9	62
C15	Kalibar	1451	38° 3ʹ 38.28″ N	46° 56ʹ 21.32″ E	12.7	401.9	62
C16	Kalibar	1453	38° 3ʹ 38.33″ N	46° 56ʹ 20.45″ E	12.7	401.9	62
C17	Kalibar	1423	38° 3ʹ 48.64″ N	46° 56ʹ 12.83″ E	12.7	401.9	62
C18	Kalibar	1431	38° 4ʹ 38.22″ N	46° 55ʹ 5.41″ E	12.7	401.9	62
C19	Kalibar	1464	38° 4ʹ 20.41″ N	46° 48ʹ 25.06″ E	12.7	401.9	62
C20	Kalibar	1461	38° 8ʹ 16.60″ N	46° 40ʹ 26.46″ E	12.7	401.9	62

### Extraction of total phenol, flavonoid, proanthocyanidin and antioxidant capacity

The fruits without seeds were powdered with liquid N. The 0.1 g of powdered sample was mixed with 5 ml of 85% methanol and vortexed for 2–3 min and was then kept at room temperature for 1 h and centrifuged at 10,000 rpm for 5 min at 4 °C. Finally, the supernatant of the samples was gently removed and stored at − 20 °C, until needed for analysis^11^.

### Determination of total phenol content (TP)

For the determination of TP, Du et al.^12^ method was used with a slight modification. First, 60 μl of the extract was mixed with 1200 μl of 10% Folin and 180 μl of distilled water. After 6 min, 960 μl of sodium carbonate was added and the final volume reached 2400 μl. The samples were kept in a dark place at room temperature for 1.5–2 h and then, the absorbance of the samples was read spectrophotometrically at 765 and the results were expressed mg GAE/100 g FW.

### Determination of total flavonoid content (TF)

Firstly, 500 μl of the prepared extract was mixed with 150 μl of 5% sodium nitrite and after 5 min, 300 μl of 10% aluminum chloride was added and one ml of 1 M NaOH was added after 5 min, then the final volume of solution was reached to 5 ml. The absorbance of samples was read spectrophotometrically at 510 nm. TF content was expressed as catechin (CAT) equivalents per 100 g FW^13^.

### Determination of total proanthocyanidin content (TPA)

For measuring TPA, BuOH–HCl assay based on the method of Cheynier et al.^14^ was used. Procyanidin dimer B1 (0.02–0.1 mg) was used to set up the standard curve (correlation coefficient, r = 0.912). TPA content was expressed as Procyanidin dimer B1 (mg Procyanidin dimer B1/100 g) equivalent.

### HPLC analysis

The samples were ground using a 1.5 ml extract solution (2:28:70, formic acid: water: methanol). The obtained extracts were shaken for 10 min in a Thermomixer (Eppendorf, USA) and were centrifuged at 10,000*g* at 4 °C. Then 0.2 μm membrane was used for filtering of 1 ml extract (Agilent Technologies, USA) for analysis.

Identification and quantification of phenolic compounds were performed according to Arabbi et al*.*^15^ using analytical reversed-phase HPLC in a Hewlett–Packard 1100 system with a quaternary pump coupled to a diode array detector. The column used was a 250 × 4.6 mm (id), 5 μm Prodigy ODS3 reversed-phase silica column (Phenomenex Ltd., Torrance, CA). The mobile phase included water: tetrahydrofuran: trifluoroacetic acid (98:2:0.1) (solvent A), and solvent B consisted of methanol: tetrahydrofuran: trifluoroacetic acid (98:2:0.1 by volume). The gradient profile was 17% B for 2 min enhancing to 25% B after 5 min, to 35% B after a further 8 min and to 50% B after a further 5 min. A column clean-up stage was performed by increasing B to 90% after a further 5 min and finally, re-equilibration was carried out for 20 min at 17% B. The flow rate and the injection volume were 1.0 ml/min and 20 μl, respectively. For identification of phenolic compounds (gallic acid, caffeic acid, chlorogenic acid, rutin, *P*-coumaric acid, rosmaric acid, quercetin, cinnamic acid, and apigenin) in the samples was used comparing their relative retention times and UV spectra with those of standard compounds and were determined utilizing an external standard method. Also, the identification and quantification of Sucrose, glucose and fructose using HPLC were done based on the Hellín et al*.*^16^ method. The elution system consisted of 0.1% phosphoric acid with a flow rate of 0.6 ml/min and was eluted through a SCR-101N column (30 cm × 9.7 mm i.d.) with an SCR (N; 5 cm × 4 mm i.d.) guard column and was detected with a RID (Shimadzu, Japan).

### Determination of antioxidant capacity

#### DPPH free radical scavenging activity

The ability of extracts to inhibit free radicals (DPPH) was measured by mixing 50 μl of the prepared extract with 950 μl of 6 × 10^−5^ mol/l methanolic DPPH solution and then samples were kept in the dark for 30 min at room temperature. The absorbance of samples was read spectrophotometrically at 517 nm. The reduction percent of DPPH was calculated by the following equation^17^:

$$ \% \,{\text{inhibition}}\,{\text{of}}\,{\text{DPPH }} = \, \left( {{\text{Abs}}\,{\text{control }} - {\text{Abs}}\,{\text{sample}}} \right)/{\text{Abs}}\,{\text{control}} \times {1}00. $$

Abs control is the absorbance of DPPH solution without the extract.

### Ferric-reducing antioxidant power (FRAP) assay

Briefly, the FRAP reagent was freshly prepared by mixing 100 mM acetate buffer (pH 3.6), 10 mM 4,6-tripryridyls-triazine (TPTZ) in 40 mM HCl and 20 mM ferric chloride in a ratio 10:1:1 (by volume) before measurement. Ten μl of the prepared extract was mixed with 3 ml of FRAP reagent and then the samples were incubated at 37 °C for 30 min. The absorbance was read by a spectrophotometer at 593 nm^18^. The absorbance of samples was read spectrophotometrically at 593 nm and the results were expressed mg Fe^2+^/100 g FW.

### Determination of vitamin C content

First, 3 ml of metaphosphoric acid was added to one gram of plant material, and then the samples were kept in a refrigerator at 4 °C. After 30 min, the samples were centrifuged at 4000 rpm for 15 min, and then the supernatant was used to measure vitamin C. The vitamin C content of samples was measured based on colour reduction of 6, 2-dichloroindophenol (DCIP) by ascorbic acid^19^. The absorbance of the samples was read at 520 nm and was expressed as mg ascorbic acid/100 g.

### Statistical analysis

This study was conducted in a completely randomized design. The data obtained were subjected to analysis of variance and means were separated by Duncan’s multiple range test at p < 0.01 significance level using SAS Software (Version 9.4). The SPSS software (Version 22) was used for correlation and the principal component analysis (PCA), and cluster analysis were performed using Stat Graphics plus 5.1 Software.

### Ethical approval

The authors confirm that the use of genotypes in the present study complies with international, national and/or institutional guidelines.

## Results and discussion

The results showed that the total phenol content of the hackberry studied genotypes had a statistically significant difference (p < 0.01, Table 2). According to the obtained results, the highest and lowest total phenol content was observed in genotype C9 (1176.73 mg GAE/100 g FW) and genotype C19 (398.27 mg GAE/100 g FW) (Table 2). Previously, Nasirifar et al.^20^ reported that the highest content of total phenol in *C.* *australis* fruits was 8.9 mg GAE/100 g DW. In another study, it was observed that the total phenol content of ripe fruit was 0.27 g GAE/100 g DW, while it was 0.05 g GAE/100 g DW in unripe fruit^4^. Also, Filali**-**Ansari et al*.*^9^ reported that the total phenol content of *C.* *australis* fruits was 286.27 mg GAE/100 g FW. Variation in the total phenol content of *C.* *australis* may be due to genotype, environmental factors, type of extraction solvent, and degree of maturity at harvest^21^.

**Table 2:** Total phenol (TP), total flavonoid (TF), total proanthocyanidin (TPA) contents, antioxidant capacity based on DPPH and FRAP assays, vitamin C and sugar contents of *Celtis* *australis* L. fruits.

G	TP (mg GAE/100 g FW)	TF (mg CAT/100 g FW	TPA (mg/100 g FW)	DPPH (%)	FRAP	Vit C (mg/100 g)	Sucrose (g/100 g)	Glucose (g/100 g)	Fructose (g/100 g)
					(mg Fe^2+^/100 g FW				
C1	1074.30 ± 0.11^b^	312.23 ± 0.01^a^	79.44 ± 0.51^a^	85.51 ± 0.31^ab^	72.60 ± 0.43^fg^	4.88 ± 0.32^k^	14.12 ± 0.88^c^	16.01 ± 0.18^bc^	18.73 ± 0.73^c^
C2	544.71 ± 0.43^f^	199.12 ± 0.03^hi^	3.00 ± 0.93^e^	72.58 ± 0.08^efg^	63.97 ± 0.31^h^	6.83 ± 0.01^d^	11.23 ± 0.07^d^	15.34 ± 0.90^c^	17.32 ± 0.15^c^
C3	1077.27 ± 0.87^b^	197.41 ± 0.18^i^	14.88 ± 0.31^cde^	64.86 ± 021^hij^	75.18 ± 0.43^fg^	5.37 ± 0.83^i^	13.43 ± 0.78^cd^	17.32 ± 0.08^b^	18.20 ± 0.32^c^
C4	505.62 ± 0.93^f^	271.02 ± 0.84^c^	7.92 ± 0.04^de^	73.20 ± 0.25^ef^	78.31 ± 0.21^efg^	6.60 ± 0.11^de^	15.34 ± 0.97^c^	18.34 ± 0.02^ab^	25.12 ± 0.01^ab^
C5	1067.11 ± 0.03^b^	198.93 ± 0.21^hi^	6.72 ± 0.01^de^	82.40 ± 0.09^k^	94.27 ± 0.43^b^	8.40 ± 0.08^b^	17.56 ± 0.05^bc^	16.12 ± 0.95^bc^	26.40 ± 0.08^ab^
C6	548.18 ± 0.05^f^	205.42 ± 0.32^fg^	2.64 ± 0.28^e^	14.12 ± 0.78^j^	93.29 ± 0.01^b^	1.12 ± 0.42^n^	11.12 ± 0.27^d^	17.09 ± 0.35^b^	19.34 ± 0.09^bc^
C7	1135.3 ± 0.76^ab^	204.51 ± 0.95^g^	17.88 ± 0.32^cde^	45.15 ± 0.42^de^	86.07 ± 0.84^b-e^	4.10 ± 0.54^l^	17.63 ± 0.68^bc^	15.34 ± 0.41^c^	24.09 ± 0.65^b^
C8	551.82 ± 0.33^f^	223.10 ± 0.54^d^	34.44 ± 0.08^c^	61.95 ± 0.97^ij^	75.72 ± 0.08^fg^	6.95 ± 0.55^fg^	19.23 ± 0.31^bc^	19.13 ± 0.18^a^	18.75 ± 0.43^c^
C9	1176.73 ± 0.97^a^	203.71 ± 0.50^fg^	26.28 ± 0.65^cd^	71.58 ± 0.90^ij^	94.05 ± 0.32^b^	7.58 ± 0.01^e^	23.67 ± 0.24^ab^	20.34 ± 0.04^a^	19.34 ± 0.51^bc^
C10	868.23 ± 0.68^d^	197.63 ± 0.88^i^	70.32 ± 0.88^ab^	88.24 ± 0.53^a^	76.48 ± 0.07^fg^	4.22 ± 0.04^m^	12.73 ± 0.68^cd^	20.98 ± 0.62^a^	25.39 ± 0.32^ab^
C11	821.24 ± 0.60^de^	214.04 ± 0.38^e^	2.40 ± 0.32^e^	75.86 ± 0.88^cde^	81.87 ± 0.32^c–f^	8.18 ± 0.08^a^	14.67 ± 0.85^c^	21.00 ± 0.31^a^	26.84 ± 0.31^ab^
C12	465.41 ± 0.59^g^	201.90 ± 0.04^ghi^	5.16 ± 0.65^de^	81.63 ± 0.39^bc^	86.07 ± 0.21^b–e^	7.50 ± 0.01^c^	25.85 ± 0.08^a^	19.43 ± 0.63^ab^	20.34 ± 0.74^bc^
C13	542.16 ± 0.38^f^	308.52 ± 0.05^a^	15.12 ± 0.33^cde^	72.89 ± 0.54^efg^	81.22 ± 0.95^def^	5.54 ± 0.18^h^	12.45 ± 0.28^cd^	18.98 ± 0.86^ab^	18.23 ± 0.84^bc^
C14	804.29 ± 0.06^de^	202.10 ± 0.66^fgh^	23.04 ± 0.98^cde^	80.49 ± 0.70^bcd^	70.12 ± 0.39^gh^	4.81 ± 0.06^m^	17.90 ± 0.91^bc^	19.87 ± 0.06^a^	26.98 ± 0.01^ab^
C15	944.65 ± 0.09^c^	196.32 ± 0.01^i^	15.24 ± 0.56^cde^	74.27 ± 0.48^de^	90.82 ± 0.05^bc^	5.00 ± 0.12^j^	21.78 ± 0.01^ab^	18.56 ± 0.04^ab^	27.04 ± 0.53^ab^
C16	554.37 ± 0.21^f^	21.59 ± 0.94^e^	19.20 ± 0.31^cde^	63.20 ± 0.38^ij^	85.75 ± 0.01^b–e^	8.11 ± 0.86^b^	18.54 ± 0.53^bc^	17.43 ± 0.08^b^	29.90 ± 0.11^a^
C17	762.07 ± 0.32^e^	313.19 ± 0.48^a^	63.36 ± 0.65^ab^	64.66 ± 0.07^hij^	79.28 ± 0.42^d–g^	7.58 ± 0.32^c^	19.43 ± 0.14^bc^	20.76 ± 0.93^a^	30.65 ± 0.54^a^
C18	775.18 ± 0.54^e^	206.82 ± 0.08^f^	24.48 ± 0.05^cd^	67.39 ± 0.02^f–i^	88.55 ± 0.86^bcd^	2.48 ± 0.64^g^	22.45 ± 0.08^ab^	17.98 ± 0.18^b^	23.43 ± 0.66^b^
C19	398.27 ± 0.76^h^	207.16 ± 0.01^f^	15.96 ± 0.93^cde^	75.45 ± 0.54^de^	44.35 ± 0.08^i^	1.96 ± 0.48^g^	13.56 ± 0.64^cd^	14.65 ± 0.84^cd^	18.09 ± 0.03^bc^
C20	966.12 ± 0.05^c^	279.24 ± 0.05^b^	17.40 ± 0.27^cde^	70.57 ± 0.87^e–h^	117.87 ± 0.07^a^	1.40 ± 0.94^ef^	20.29 ± 0.42^b^	18.82 ± 0.31^ab^	24.84 ± 0.43^b^

Values in the same column with different lowercase letters are significantly different at p < 0.01.

The differences in the total flavonoid content of the *C.* *australis* studied genotypes were statistically significant (p < 0.01, Table 2). According to the results, the highest total flavonoid content was 313.19 mg CA/100 g FW, which was observed in genotype C17. While the lowest content of total flavonoid was observed in genotype C3 (197.41 mg CA/100 g FW) (Table 2). Previously, it was reported that the total flavonoid content of *C.* *australis* was 97.69 mg quercetin/g FW^9^. Furthermore, in Nasirifar et al*.*^20^ study, the total flavonoid content of *C.* *australis* fruits was found 3.34 mg quercetin/g DW.

The results of the present study revealed that differences in the total proanthocyanidin (TPA) content of *C.* *australis* fruits were statistically significant (p < 0.01, Table 2). The highest and lowest total TPA content among the *C.* *australis* studied genotypes were 79.44 mg/100 g (genotype C1) and 2.40 mg/100 g (Genotype C11) (Table 2). The TPA levels in the present study were comparable to those reported in *Celtis* *tournefortii* fruits by Keser et al*.*^22^, which showed the TPA content of *Celtis* *tournefortii* fruits in water, ethanol, and methanol extracts were 187.44, 154.11, 265.22 μg CE/g extract, respectively.

The differences in the sugar content were statistically significant (p < 0.05, Table 2) in all of the genotypes. According to the results of the present study, the range of sucrose, glucose, and fructose content was 11.12–25.85, 14.65–21.00 and 17.32–30.65 g/100 g, respectively (Table 2). These results are in agreement with the Vidal-Cascales et al*.*^23^ study, which reported the content of sucrose, glucose, and fructose was 12.8, 17.5 and 21.8 g/100 g in the flash of *C.* *australis*, respectively. While sucrose, glucose, and fructose content of *C.* *australis* peel were 14.5, 15.4, and 18.9 g/100 g, respectively.

The differences in vitamin C content among twenty *C.* *australis* genotypes were statistically significant (p < 0.01, Table 2). Based on the results, genotype C11 (8.18 mg ascorbic acid/100 g) had the highest content of vitamin C and genotype C6 (1.12 mg ascorbic acid/100 g) had the lowest content of vitamin C among the studied genotypes. Vidal-Cascales et al*.*^23^ revealed that the content of ascorbic acid in flash and peel was 2.2 mg/100 g and 3.7 mg/100 g, respectively. In another study, the vitamin C content of Algerian hackberry fruits was recorded at 3.9 mg/100 g^24^. Our results were higher than the results of the previous studies mentioned above which may be due to different environmental conditions, genotypes and type of extraction solvent.

In the present study, the antioxidant capacity of *C.* *australis* studied genotypes was evaluated by DPPH and FRAP methods. Increasing attention to food therapy and foods with therapeutic and medicinal properties has led to the beginning of plant breeding to select the genotypes with higher levels of antioxidant compounds^25^. The differences in antioxidant capacity based on DPPH and FRAP assays among all *C.* *australis* genotypes were statistically significant (p < 0.01, Table 2). Based on the obtained results, the highest antioxidant capacity of *C.* *australis* fruits based on DPPH and FRAP assays was observed in genotypes C10 (88.24%) and C20 (117.87 mg Fe^2+^/100 g). While the lowest values were observed in genotypes C6 (14.12%) and C19 (44.35 mg Fe^2+^/100 g) (Table 2). Our results are very close to those of Nasirifar et al*.*^20^ who observed that the antioxidant capacity of *C.* *australis* fruits based on DPPH was 63.45%. Furthermore, Ota et al.^4^ found that the antioxidant activity of *C.* *australis* fruits was higher in aqueous extract and a positive and significant correlation between cyanidin-3,5-di-*O*-glucoside and vanilic acid compounds with antioxidant capacity at the end of the growing season was observed. In recent years, interest in fruits with high antioxidant capacity has increased, which according to our results, *C.* *australis* fruit is a good source of antioxidants.

Individual phenolic compounds were recognized by HPLC in the fruit of all studied *C.* *australis* genotypes. Gallic acid, caffeic acid, chlorogenic acid, rutin, *p*-coumaric acid, rosmaric acid, quercetin, cinnamic acid, and apigenin were detected and quantified in the fruits (Table 3). Based on the results, gallic acid was not identified in C2, C4, C7, C19 and C20 genotypes, but in the other genotypes, the gallic acid content varied between 2.59 and 26.32 mg/g. Previously, Sommavilla et al.^26^ revealed that *C.* *australis* contained caffeic acid and its derivatives. The content of caffeic acid ranged from 2.03 to 9.32 mg/g. While the content of chlorogenic acid in *C.* *australis* genotypes ranged from 0.94 to 11.35 mg/g. Previously, it was found that chlorogenic acid as a common phenolic acid in different plants has antioxidant and antitumor potential and it plays an important role in the synthesis of flavour compounds^27^. The results showed that the rutin content of *C.* *australis* genotypes varied between 1.80 and 4.857 mg/g. It has been found that rutin has antioxidant and antimicrobial properties and is related to other beneficial health processes^28^. Also, our results showed that the *p*-coumaric acid content of *C.* *australis* fruits ranged from 2.32 to 9.52 mg/g, while the rosmaric acid content varied between 4.74 and 51.38 mg/g.

**Table 3:** Individual phenolic compounds of *Celtis* *australis* fruits in studied genotypes (mg/g) determined by HPLC.

G	Gallic	Caffeic	Chlorogenic	Rutin	*p*-coumaric	Rosmaric	Quercetin	Cinamic	Apigenin
C1	26.32 ± 1.48^a^	2.84 ± 0.75^cd^	2.33 ± 0.43^d^	2.59 ± 0.15^b^	4.81 ± 0.31^c^	10.42 ± 0.08^e^	2.00 ± 0.01^bc^	0.21 ± 0.48^d^	0.55 ± 0.54^cd^
C2	N.D	4.34 ± 0.43^bc^	11.35 ± 0.28^a^	2.18 ± 0.17^ab^	3.58 ± 0.03^cd^	11.46 ± 0.13^de^	2.07 ± 0.08^bc^	0.18 ± 0.36^de^	0.53 ± 0.13^cd^
C3	4.38 ± 1.43^d^	4.92 ± 0.01^bc^	2.14 ± 0.84^d^	3.78 ± 0.32^ab^	4.01 ± 0.04^c^	18.81 ± 0.09^cd^	4.02 ± 0.10^ab^	0.42 ± 0.28^c^	1.06 ± 0.04^b^
C4	N.D	2.37 ± 0.33^cd^	5.57 ± 0.73^c^	2.30 ± 0.17^b^	2.43 ± 0.10^d^	13.25 ± 0.17^de^	2.46 ± 0.33^bc^	0.24 ± 0.18^d^	0.65 ± 0.05^c^
C5	2.59 ± 0.91^e^	2.07 ± 0.09^cd^	1.04 ± 0.41^e^	1.80 ± 0.09^bc^	4.20 ± 0.20^c^	10.23 ± 0.32^e^	1.86 ± 0.24^c^	0.21 ± 0.02^d^	0.51 ± 0.95^cd^
C6	13.40 ± 0.65^b^	2.49 ± 0.54^cd^	4.74 ± 0.23^ cd^	2.04 ± 0.03^ab^	2.44 ± 0.01^d^	14.73 ± 045^d^	2.27 ± 0.277^bc^	0.25 ± 0.27^d^	0.66 ± 0.29^c^
C7	N.D	3.31 ± 0.33^c^	1.31 ± 0.34^e^	2.15 ± 0.94^b^	2.60 ± 0.24^d^	10.10 ± 0.24^e^	2.15 ± 0.01^bc^	0.31 ± 0.38^cd^	0.58 ± 0.27^cd^
C8	4.26 ± 0.99^d^	6.98 ± 0.23^b^	10.02 ± 0.28^a^	4.57 ± 0.32^a^	5.50 ± 0.32^bc^	22.32 ± 0.48^c^	4.24 ± 0.06^ab^	0.48 ± 0.64^c^	1.26 ± 0.19^ab^
C9	5.46 ± 1.33^d^	3.03 ± 0.77^c^	1.31 ± 0.91^e^	2.05 ± 0.32^b^	9.52 ± 0.05^a^	8.90 ± 0.12^f^	2.22 ± 0.32^bc^	0.19 ± 0.26^de^	0.56 ± 0.04^cd^
C10	10.09 ± 0.68^c^	3.17 ± 0.87^c^	1.43 ± 0.22^e^	2.04 ± 0.45^b^	2.92 ± 0.91^d^	11.39 ± 0.15^de^	1.86 ± 0.64^c^	0.21 ± 0.84^d^	0.88 ± 0.17^bc^
C11	3.14 ± 0.92^de^	2.61 ± 0.09^cd^	1.36 ± 0.65^e^	3.28 ± 0.76^ab^	3.17 ± 0.54^cd^	12.53 ± 0.83^de^	2.40 ± 0.28^bc^	0.30 ± 0.34^cd^	0.71 ± 0.81^bc^
C12	14.14 ± 0.88^b^	4.25 ± 0.96^bc^	6.21 ± 0.44^bc^	3.56 ± 0.08^ab^	6.34 ± 0.23^b^	42.01 ± 0.10^b^	4.39 ± 0.10^ab^	0.43 ± 0.10^c^	1.26 ± 0.29^ab^
C13	3.74 ± 0.93^de^	2.03 ± 0.32^cd^	1.43 ± 0.94^e^	1.93 ± 0.32^bc^	4.07 ± 0.45^c^	16.70 ± 0.02^cd^	1.73 ± 0.34^c^	0.29 ± 023^cd^	1.01 ± 0.25^b^
C14	5.87 ± 1.08^d^	9.32 ± 0.75^a^	7.91 ± 0.11^b^	4.27 ± 0.17^a^	2.97 ± 0.08^d^	51.38 ± 0.17^a^	6.04 ± 0.28^a^	1.42 ± 0.64^b^	1.55 ± 036^a^
C15	3.67 ± 1.03^de^	5.03 ± 0.43^bc^	7.91 ± 0.04^b^	4.12 ± 018^a^	5.12 ± 0.06^bc^	18.44 ± 0.58^cd^	4.71 ± 0.17^ab^	0.39 ± 0.37^c^	1.37 ± 0.18^ab^
C16	3.05 ± 2.03^de^	4.79 ± 0.09^bc^	7.74 ± 0.93^b^	2.33 ± 0.38^b^	2.70 ± 0.33^d^	12.36 ± 0.28^de^	2.61 ± 0.19^bc^	0.21 ± 0.78^d^	0.90 ± 0.05^b^
C17	5.78 ± 0.88^d^	3.06 ± 0.05^c^	0.94 ± 0.02ef	2.09 ± 0.52^b^	2.32 ± 0.13^d^	9.66 ± 0.03^ef^	1.73 ± 0.60^c^	0.22 ± 0.38^d^	0.52 ± 0.16^cd^
C18	13.64 ± 0.98^b^	2.10 ± 0.02^cd^	1.69 ± 0.55^e^	2.04 ± 0.63^b^	2.40 ± 0.19^d^	4.74 ± 0.09^g^	1.05 ± 0.18^ cd^	2.10 ± 0.12^a^	0.27 ± 0.13^d^
C19	N.D	2.38 ± 0.04^cd^	7.08 ± 0.35^b^	1.91 ± 0.15^bc^	6.32 ± 0.13^b^	9.57 ± 0.35^ef^	1.99 ± 0.95^c^	0.19 ± 0.19^de^	0.86 ± 0.44^b^
C20	N.D	3.80 ± 0.33^c^	6.20 ± 0.18^bc^	3.40 ± 0.19^ab^	4.57 ± 0.75^c^	21.45 ± 0.13^c^	3.30 ± 0.04^b^	0.40 ± 0.09^c^	1.18 ± 0.12^ab^

Values in the same column with different lowercase letters are significantly different at p < 0.05.

The quercetin content of *C.* *australis* fruits in all studied genotypes varied between 1.05 and 6.04 mg/g, while the cinamic acid and apigenin content of *C.* *australis* fruits ranged from 0.18–2.10 and 0.27–1.37 mg/g, respectively. In general, the results revealed that the phenolic compositions of *C.* *australis* fruits were genotype-dependent and the differences in the values obtained among *C.* *australis* genotypes might be resulting from the edaphoclimatic conditions. Keser et al*.*^22^ identified rutin (0.55 µg/g), morin (0.05 µg/g), quercetin (0.05 µg/g), kaempferol (0.05 µg/g), naringin (0.35 µg/g), naringenin (0.05 µg/g), resveratrol (0.05 µg/g), vanillic acid (29.10 µg/g), gallic acid (32.95 µg/g), caffeic acid (5.90 µg/g), ferulic acid (19.45 µg/g) and rosmarinic acid (1.20 µg/g). in *C.* *tournefortii* fruits. Also, in a previous study by Gecibesler^29^ on *C.* *tournefortii*s, it was found that the predominant phenolic compounds in leaf, fruit and young twig included fumaric acid, gentisic acid, vanilic acid, and scutellarin. In another study, phenolic compounds such as caffeic acid (0.756 mg/g), chlorogenic acid (6.882 mg/g), p-coumaric acid (1.968 mg/g), rutin (8.661 mg/g), ellagic acid (12.783 mg/g), catechin (34.821 mg/g), myricetin (3.015 mg/g) were reported in fruit of *C.* *tournefortii*^30^. Several factors such as cultivars, climate and region (north or south), cultivation method (greenhouse or outdoors), farming style (conventional or organic), crop load, time of harvest, soil and certain detection methodologies were affected the contents of phenolic compounds^31^.

The obtained results of correlation analysis could be utilized in breeding programs^32^. The results of the Pearson correlation between phenolic compounds and antioxidant assays for twenty genotypes of *C.* *australis* are shown in Table 4. It was found that the TP (r = 0.718), gallic acid (r = 0.673), chlorogenic acid (r = 0.714), *p*-comaric (r = 0.587), and apigenin (r = 0.485) phenolic compounds had a high positive correlation with FRAP assay, which demonstrate their high contribution to the antioxidant capacity based on FRAP assay in *C.* *australis* genotypes. While TF (r = 0.405) and quercetin (r = 0.414) revealed a moderate and significant correlation with FRAP assay. Previously, Ozgen et al*.*^33^ in black raspberries fruits, Alvarez et al*.*^34^ in apple beverages and De Sousa et al*.*^35^ in *Oenocarpus* *distichus* mart fruits revealed that TP, TF, chlorogenic acid and rutin had a high correlation with antioxidant capacity properties. Also, the results of the correlation showed that TPA (r = 0.596) had a high positive correlation with the DPPH assay. While rutin (r = 0.339) and rosmaric acid (r = 0.374) presented a moderate and significant correlation with the DPPH assay.

**Table 4:** Pearson’s correlation coefficients between phenolic compounds and antioxidant assays in different genotypes fruit of *Celtis* *australis* L.

	DPPH	FRAP
TP	0.130^ns^	718**
TF	0.086^ns^	0.405*
TPA	0.596**	− 0.160^ns^
Vit c	0.046^ns^	− 0.056^ns^
Gallic	0.032^ns^	0.673**
Caffeic	0.091^ns^	− 0.140^ns^
Chlorogenic	− 0.020^ns^	0.714**
Rutin	0.339*	0.066^ns^
*p*-coumaric	0.021^ns^	0.587**
Rosmaric	0.374	− 0.002^ns^
Quercetin	0.152^ns^	0.414*
Cinamic	0.047^ns^	0.152^ns^
Apigenin	0.008^ns^	0.485**

ns, no significant; *significant at p ≤ 0.05; **significant at p ≤ 0.001. Parameters: *TP* total phenol, *TF* total flavonoid, *TPA* total proanthocyanin, *DPPH* DPPH assay, *FRAP* FRAP assay, *Vit* *c* vitamin C.

**Figure 1:**
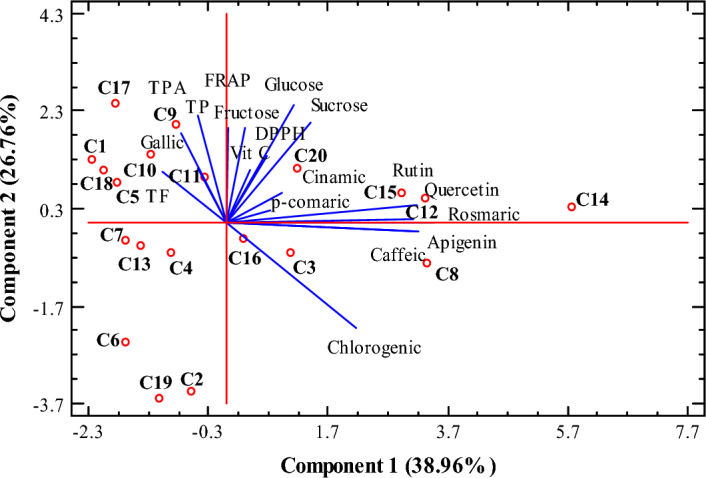
Principal component analysis of genotypes and studied parameters of *Celtis* *australis*. *TP* Total phenol, *TF* Total flavonoid, *TPA* total proanthocyanin, *Vit* *C* vitamin C, *FRAP* FRAP assay, *DPPH* DPPH assay.

The principal component analysis (PCA) was performed to assess and determine the variation between the phenolic and antioxidant compounds based on the studied genotypes. According to the results, the first two PCA explain 65.52% (38.96% and 26.76%, respectively) of all variance for *C.* *australis* genotypes (Fig. 1.). As it can be shown, the PC1 was positively linked to caffeic acid, chlorogenic acid, rutin, *p*-coumaric acid, rosmaric acid, quercetin, cinnamic acid, and apigenin. While, PC2 was positively correlated with gallic acid, TP, TF, and TPA. All the *C.* *australis* genotypes are plotted on the reduced space of the two first principal components.

As shown in Fig. 1, the C12, C14, C15, C20, C8, C16, C3, and C20 genotypes are positively characterized by PCA1. Thus, these genotypes have a higher content of caffeic acid, chlorogenic acid, rutin, *p*-coumaric acid, rosmaric acid, quercetin, cinnamic acid, and apigenin that might be good candidates in breeding programs to increase phenolic compounds. On the contrary, the C2, C4, C6, C13, C7, and C19 genotypes have lower content of phenolic and antioxidant compounds, and sugar content, which formed a group with negative values for both PC1 and PC2. Therefore, these genotypes are less considered in terms of nutritional value. The C5, C10, C11, C1, C18, C17, and C9 genotypes are positively characterized by the second principal component, which has a higher content of gallic acid, TP, TF, and TPA. Therefore, PCA could be helpful to present valuable information on the relationship between phenolic and antioxidant compounds in all studied genotypes.

**Figure 2:**
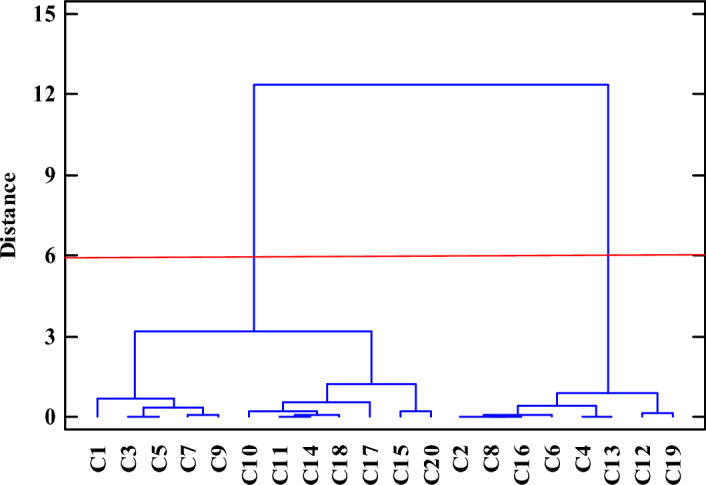
Dendrogram of 20 *Celtis* *australis* L. genotypes based on Ward's method.

Cluster analysis was carried out to investigate the similarities and differences among all studied genotypes. In the present study, all studied genotypes were grouped using Ward's method based on all measured traits. The *C.* *australis* genotypes were divided into 2 main clusters (Fig. 2). Also, the first cluster was divided into 2 subgroups which C1, C3, C5, C5 and C9 genotypes were located in the first subgroup which all of them related to the Khodaafarin region, while in the second subgroup, 7 genotypes (C10, C11, C14, C18, C17, C15 and C20) were located, which the all of them except C10 related to Kalibar region. In the second cluster, 8 genotypes (C2, C8, C16, C6, C4, C13, C12 and C19) were located. Therefore, the presence of genotypes in different groups revealed high diversity among all studied genotypes based on the evaluated traits. So, the genotypes located in the first subgroup and second cluster having greater distance can be utilized in the future breeding program. In general, it can be concluded that the grouping of all studied *C.* *australis* genotypes resulted from environmental conditions.

## Conclusion

The results showed that the studied *C.* *australis* genotypes contained different polyphenols compounds. As *C.* *australis* fruits are widely consumed in some countries such as Iran, Turkey, etc., the present study revealed that *C.* *australis* fruits have promising compounds for further research and made a high-quality derived food product with high health benefits to better the human life-quality. The *C.* *australis* wild genotypes studied in the Arasbaran region are valuable genetic resources in terms of the ability to produce phenolic and antioxidant compounds. Therefore, the studied genotypes can be used to create cultivars rich in phenolic and antioxidant compounds via hybridization.

## Data Availability

The datasets used and/or analyzed during the current study are available from the corresponding author upon reasonable request.
